# The relationship between total tau protein, phosphorylated tau protein, schizophrenia and bipolar disorder: a systematic review and meta-analysis

**DOI:** 10.3389/fpsyt.2026.1764148

**Published:** 2026-03-05

**Authors:** Sien Hou, Lingxi Chen, Linxin Chang, Chunyue Huo

**Affiliations:** 1 Capital Medical University, Yanjing Medical College, Beijing, China; 2 School of Education, The University of New South Wales (UNSW), Sydney, NSW, Australia

**Keywords:** schizophrenia, meta-analysis, bipolar disorder, tau phosphorylation, total tau (t-tau)

## Abstract

**Introduction:**

Neurodevelopmental disorders, notably schizophrenia, continue to pose major challenges in mental health and clinical practice. Numerous studies have posited potential associations between tau proteins and schizophrenia or bipolar disorder (BD), yet these associations have not been systematically described or quantitatively examined. This study aims to compare total tau and phosphorylated tau levels in plasma, serum (collectively referred to as peripheral blood), and cerebrospinal fluid (CSF) between individuals with schizophrenia and healthy controls, and to further examine total tau levels in BD.

**Methods:**

Employing a meticulous search strategy across PubMed, Embase, Medline and Web of Science, this study adheres to PRISMA guidelines. Eligible studies were non-randomized controlled trials investigating associations between tau proteins, schizophrenia or BD. Standardized mean differences (SMDs) with 95% confidence intervals (CIs) were calculated. This study protocol has been registered on PROSPERO (CRD420251123530).

**Results:**

10 studies were included in the meta-analysis. Total tau levels were significantly lower in patients with schizophrenia compared to controls in CSF (SMD = -0.33, 95% CI: [-0.59, -0.08]) and peripheral blood samples (SMD = -0.88, 95% CI: [-1.61, -0.15]). Combining both samples, the overall SMD was -0.48 (95% CI: [-0.66, -0.31]), indicating a significant reduction in total tau levels. The pooled analysis for phosphorylated tau group yielded an SMD of -1.07 (95% CI: [-1.55, -0.59]), with consistent findings in CSF samples (SMD = -0.76, 95% CI:[-1.17, -0.35]). In contrast, total tau levels did not differ significantly between patients with BD and healthy controls (SMD = -0.07, 95% CI: [-0.26, 0.12]), Data were insufficient to support a meta-analysis of the relationship between phosphorylated tau and BD.

**Conclusion:**

T-tau and p-tau protein levels are lower in schizophrenia patients compared with healthy controls, whereas no significant difference was observed in BD patients. These findings may have potential for clinical diagnostics.

**Systematic Review Registration:**

https://www.crd.york.ac.uk/prospero/, identifier CRD420251123530.

## 1 Introduction

Severe mental disorders, including schizophrenia (SCZ) and bipolar disorder (BD), pose substantial public health challenges due to their chronic course, functional impairment, and limited biomarker-supported diagnostic tools. Schizophrenia is among the most debilitating mental disorders worldwide (1), whereas BD increases the mortality rate and diagnostic difficulty (2). Tau protein, a microtubule-associated and heat-stable protein predominantly located in axons of neurons, plays a crucial role in microtubule assembly (3). It is also a well-established biomarker for Alzheimer’s disease (4, 5). In patients with Alzheimer’s disease, tau is excessively or abnormally phosphorylated (6), aggregating within neurons to form neurofibrillary tangles, reducing the stability of microtubules, and leading to neuronal degeneration. This pathological process not only triggers Alzheimer’s disease but may also lead to other neurodegenerative diseases, collectively termed tauopathies (7). Notably, some studies have pointed out that schizophrenia may be a type of tauopathy (8). However, certain previous neurobiological studies have not supported the neurodegenerative theory of schizophrenia (9). This contradiction underscores a key discrepancy that our study aims to clarify. There are currently no confirmed biomarkers for schizophrenia and BD, and no meta-analyses on the relationship between these two diseases and tau protein. By integrating data from various studies, this meta-analysis aims to clarify the associations between total tau and phosphorylated tau levels and the diagnoses of schizophrenia and BD. Given the absence of established biomarkers for these disorders, elucidating tau-related changes may offer novel insights for diagnosis and pathophysiology.

## 2 Methods

### 2.1 Study registration and reporting standards

This study adhered to PRISMA guidelines. No restrictions were imposed with respect to language, publication date, or publication status. The protocol has been registered on PROSPERO (CRD420251123530), and detailed registration information can be obtained online.

### 2.2 Search strategy

A comprehensive literature search was performed up to August 25, 2025, using PubMed, Embase, Web of Science, and Medline. Both controlled vocabulary and keywords were used, including “schizophrenic disorder”, “dementia praecox”, “microtubule-associated protein tau”, and “MAPT”. The number of retrieved articles on BD and phosphorylated tau protein was insufficient to support a quantitative synthesis.

### 2.3 Eligible criteria

Inclusion criteria: (1) The study utilized individuals diagnosed with schizophrenia or BD (first episode or chronic) according to the DSM-V or ICD-10 criteria or Hamilton Depression Scale (HAMD), with healthy control groups; (2) Studies were excluded if there were known major confounding factors affecting tau levels of total tau protein and phosphorylated tau protein in either the experimental group or the control group, and no special intervention measures were taken; and (3) results must include direct or indirect measurements of the total tau protein level and phosphorylated tau protein level.

Exclusion criteria: In the first selection stage (title and abstract), the exclusion criteria were applied in the following order: (1) Review, meta-analysis or conference abstract; (2) Unrelated research; (3) Non-human studies; (4) Withdrawn research. In the second stage (full text), the exclusion criteria are applied in the following order: (1) No control experiments with healthy people were set up. (2) Essential outcome data could not be obtained.

### 2.4 Data extraction

Data extraction was conducted directly from the full text by two independent reviewers (H.S. and C.L.) To address any discrepancies, a third reviewer (Z.B.) was consulted.

Two independent reviewers extracted data using EndNote21, extracted information included study characteristics, tau measurement site, assay type, demographic details, and tau concentration metrics. When necessary, authors were contacted; otherwise, WebPlotDigitizer was used to estimate numeric values from graphs.

The retrieved literature was imported into EndNote21 for data extraction. If the article did not provide relevant data or research information, the original author was contacted to obtain the data. If the original author did not reply, the digital ruler WebPlotDigitizer was used to estimate the axis data. Subsequent statistical methods were adopted to calculate the mean and standard deviation. Each article extracted the title, author, year, sample size, sample gender, sample age, total tau protein concentration of the sample, measurement method of total tau protein concentration, extraction site, sample type, whether follow-up was conducted, and research conclusion. Data from each study presented the results of each comparison in the form of mean ± standard deviation (SD) to ensure uniformity and standardization of the included data.

### 2.5 Methodological quality assessment

As this study was an observational study, the review authors evaluated the risk of bias based on the Newcastle-Ottawa Scale (NOS), which evaluated across three dimensions: subject selection, comparability, and outcome measurement. Additionally, corresponding scores were given.

### 2.6 Statistical analysis

Statistical significance of the results was set at a two-sided p-value of p < 0.05, which indicates that the results are meaningful. And when the CI range does not include zero, the result was considered statistically significant. Due to the differences in research design and study population, there might be significant differences among studies. Therefore, a random-effects model was adopted, Review Manager software was used to evaluate heterogeneity, and I² statistics were supplemented. To assess the possibility of publication bias in research, a bias funnel plot was used, and publication bias was evaluated by the symmetry of the funnel plot. Considering the significant heterogeneity among different studies, we adopted SMD as the effect indicator for the analysis. The presentation of data includes effect size and 95% CI, providing a forest chart for comparing effect size. Analyses were conducted using Review Manager 5.4.1 to ensure accuracy.

## 3 Results

### 3.1 Search results

**Figure 1:**
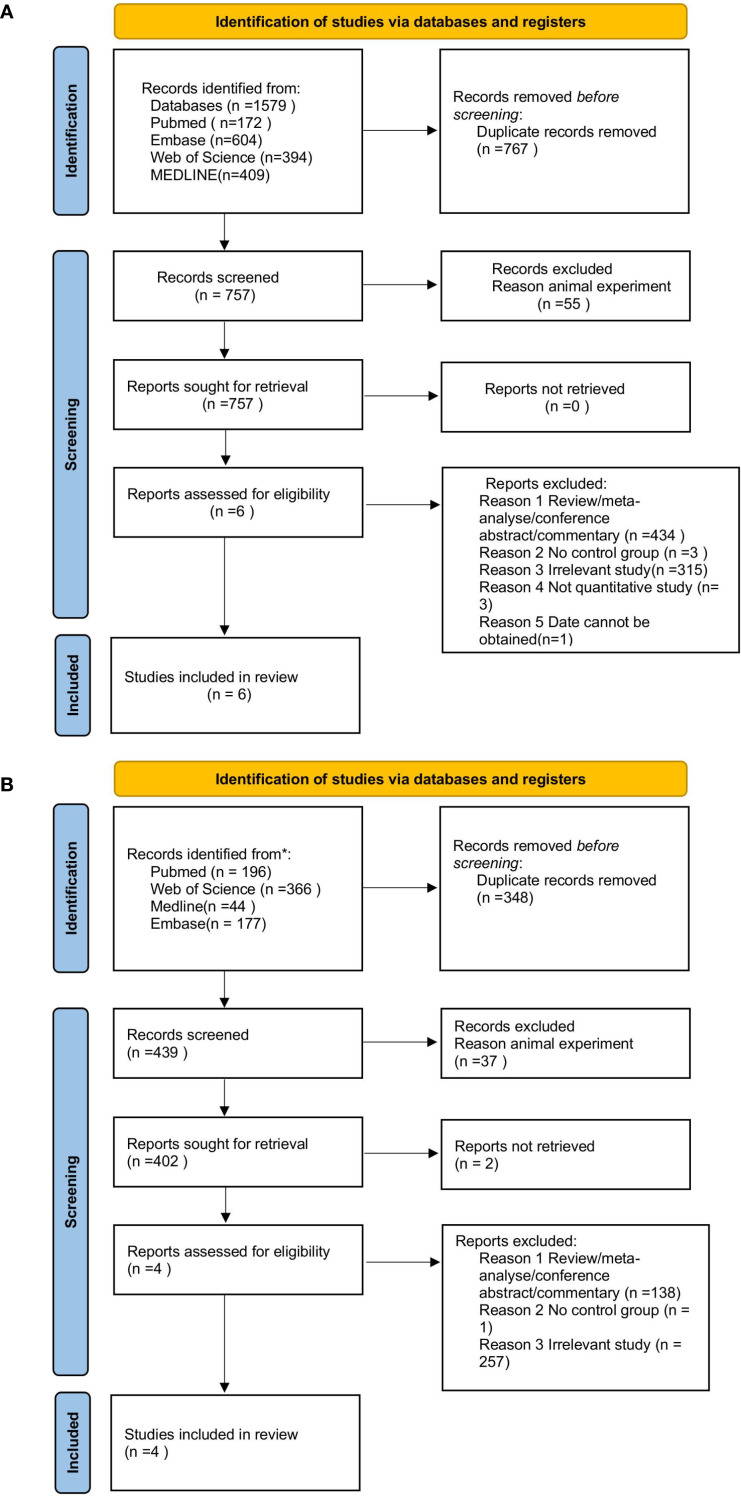
**(A)** PRISMA flow-diagram of the study selection process. (schizophrenia). **(B)** PRISMA flow-diagram of the study selection process. (bipolar disorder).

Ten studies met the inclusion criteria and were included in this study (8–12, 12–16), (**Tables 1**–**3**), and detailed data extraction processes were carried out for each article (**Figures 1A, B**). The included studies had a wide coverage, ensuring a comprehensive analysis of the impact of total tau protein on schizophrenia and BD.

**Table 1:** The main characteristics of the 6 studies included in the schizophrenia group (2003-2025) are based on the Newcastle-Ottawa Quality Assessment criteria (t-tau).

References; year of publication	Country	Study design	Sample	Mean age; male	Diagnostic criteria for schizophrenia	The total tau protein levels of the control group/patient group	Selection site of tau protein	Quality assessment (Newcastle-Ottawa Score)
Andreou et al., (10)	UK	Case–control study	Control:55 Patient:29	Control:16.2;46.8% Patient:16.4;29.7%	DSM-V	Control:9965(1137)/10160(1106) Patient:9345(958)/9672(1057)	plasma	Selection:3 Comparability:2 Outcome:1
Schönknecht et al. (9)	German	Case–control study	Control:10 Patient:9/10	Control: 65.5/37.7;4/5 Patient: 64.2/27.7;3/7	DSM-V	Control:217(66.1)/172.2(76.3) Patient:190.6(109)/178(101.6)	CSF	Selection:3 Comparability:2 Outcome:1
Frisoni et al., 2011 (11)	Italy	Case–control study	Control:6 Patient:11	Control:61.3;5 Patient:69.2;3	DSM-V	Control:255(133) Patient:171(51)	CSF	Selection:3 Comparability:3 Outcome:1
Demirel et al. (8)	Turkey	Case–control study	Control:42 Patient:42	Control:36.6;22 Patient:39.1;21	DSM-V	Control:270.4(117.19) Patient:100.2(85.13)	serum	Selection:3 Comparability:2 Outcome:1
Petra et al., 2025 (12)	German	Case–control study	Control:29 Patient:54	Control:46.5;9 Patient:34.4;32	ICD-10	Control:263(91) Patient:208(104)	CSF	Selection:4 Comparability:1 Outcome:2
Runge et al. (13)	German	Case–control study	Control:39 Patient:100	Control:34.62;6 Patient:33.72;40	ICD-10	Control:151.14(73.27) Patient:138.33(61.04)	CSF	Selection:3 Comparability:1 Outcome:1

(The following article strictly controls other neurological diseases of the patient: (10, 13), (12), (8).

The following article strictly controls the use of psychotropic drugs by patients: Andreou et al.2021, (11). It is worth noting that all articles exclude patients with Alzheimer’s disease)

**Table 2:** The main characteristics of the 4 studies included in the bipolar disorder group (2013-2024) are based on the Newcastle-Ottawa Quality assessment criteria.

References; year of publication	Country	Study design	Sample	Mean age; male	Diagnostic criteria for bipolar disorder	The tau protein levels of the control group/patient group	Selection site of tau protein	Quality assessment (Newcastle-ottawa score)
Ulla et al., 2024 (14)	Denmark	Case–control study	Control:44 Patient:85	Control:30.5;25 Patient:33;44	HAMD	Control:195.61(73.25) Patient:205.3(82.05)	CSF	Selection:4 Comparability:2 Outcome:2
Forlenza et al. (15)	Brazil	Case–control cstudy	Control:25 Patient:16	Control:72;5 Patient:70.5;4	DSM-V	Control:83.6(51.5) Patient:90.4(67.2)	CSF	Selection:4 Comparability:1 Outcome:1
Sindre et al., 2015 (16)	Sweden	Case–control study	Control:71 Patient:82	Control:37.8;27 Patient:38.3;34	DSM-V	Control:37.17(14.09) Patient:34.32(12.48)	CSF	Selection:4 Comparability:1 Outcome:1
Joel et al., 2013 (14)	Sweden	Case–control study	Control:69 Patient:84	Control: None; Patient: None;55	DSM-V	Control:36(14) Patient:35(14)	CSF	Selection:4 Comparability:1 Outcome:1

**Table 3:** The main characteristics of the 5 studies included in the schizophrenia group (2003-2025) are based on the Newcastle-Ottawa Quality Assessment criteria (p-tau).

References; year of publication	Country	Study design	Sample	Mean age; male	Diagnostic criteria for bipolar disorder	The phosphorylated tau protein levels of the control group/patient group	Selection site of tau protein	Quality assessment (Newcastle-ottawa score)
Frisoni et al., 2011 (11)	Italy	Case–control study	Control:6 Patient:11	Control:61.3;5 Patient:69.2;3	DSM-V	Control:49(28) Patient:32(8)	CSF	Selection:3 Comparability:3 Outcome:1
Runge et al. (13)	German	Case–control study	Control:39 Patient:100	Control:34.62;6 Patient:33.72;40	ICD-10	Control:27.59(11.74) Patient:18.03(8.01)	CSF	Selection:3 Comparability:1 Outcome:1
Demirel et al. (8)	Turkey	Case–control study	Control:42 Patient:42	Control:36.6;22 Patient:39.1;21	DSM-V	Control:160.3(73.34) Patient:78.7(37.99)	serum	Selection:3 Comparability:2 Outcome:1
Schönknecht et al. (9)	German	Case–control study	Control:10 Patient:9/10	Control:65.5/37.7;4/5 Patient: 64.2/27.7;3/7	DSM-V	Control:46.4(8.8)/38(11.6) Patient:43.3(17.9)/38.5(14.4)	CSF	Selection:3 Comparability:2 Outcome:1
Petra et al., 2025 (12)	German	Case–control study	Control:29 Patient:54	Control:46.5;9 Patient:34.4;32	ICD-10	Control:34(9.7) Patient:24(9)	CSF	Selection:4 Comparability:1 Outcome:2

### 3.2 Outcome

**Figure 2:**
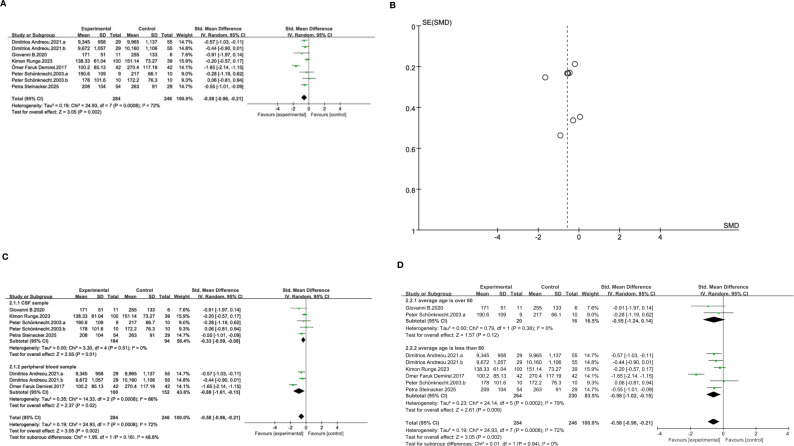
**(A)** Forest plot of SMD and 95% CIs for t-tau and schizophrenia. **(B)** Funnel plot for main analysis for t-tau and schizophrenia. **(C)** Forest plot of Std Mean Difference and 95% CIs for t-tau in cerebrospinal fluid and peripheral blood of patients with schizophrenia. **(D)** Forest plot of Std Mean Difference and 95% CIs for t-tau in patients with average age over 60 and average less than 60.

To accurately investigate the association between total tau protein and schizophrenia, we conducted a meta-analysis of six studies. In the studies, the diagnostic criteria for schizophrenia were DSM-V, ICD-10 and HAMD. Our meta-analysis found that the standardized mean difference (SMD) between schizophrenia and total tau protein was -0.58 (95% CI: [-0.96, -0.21], p=0.0008) (**Figure 2A**), and the confidence interval did not include 0, which was statistically significant (p=0.0008). Although the overall symmetry of the funnel plot is relatively poor (**Figure 2B**), this higher heterogeneity may be attributed to different sample types (using plasma or CSF as samples). Another point that needs to be emphasized is that the chart distinguishes between the orbitofrontal lobe (a) and the precentral gyrus (b) in Dimitrios’ study, and between participants over 50 years old (a) and those under 50 years old (b) in Peter’s study. The results of the comprehensive meta-analysis show that the total tau protein concentration in patients with schizophrenia is lower than that in the control group.

Subgroup analyses were conducted based on sample type and average age (**Figures 2C, D**). We define studies that use serum and plasma as samples as peripheral blood samples, while all the rest of the studies adopt cerebrospinal fluid samples. The results show that based on cerebrospinal fluid samples, the total tau of schizophrenia patients decreased, and it is statistically significant (SMD: -0.33, 95% CI: [-0.59, -0.08], p=0.01), and the cerebrospinal fluid samples have extremely low heterogeneity. Although the peripheral blood subgroup also showed a significant reduction in total tau, the heterogeneity of the studies is relatively high and the peripheral blood subgroup also showed a significant reduction (SMD: -0.88, 95% CI: [-1.61, -0.15], p=0.002). These results indicate that the determination of total tau using cerebrospinal fluid samples is more robust than that using peripheral blood samples.

Based on the World Health Organization’s definition of the elderly, we define patients with an average age of over 60 as elderly patients and those under 60 as young patients. The conclusion of the subgroup of elderly patients was not statistically significant (SMD=-0.55, 95%CI: [-1.24, 0.14], p=0.12), and due to the limited sample size, it could not indicate a significant decrease in total tau in elderly patients. However, the total tau in young patients showed a significant downward trend (SMD=-0.58, 95%CI: [-1.02, -0.15], p=0.009), but there was still some heterogeneity.

**Figure 3:**
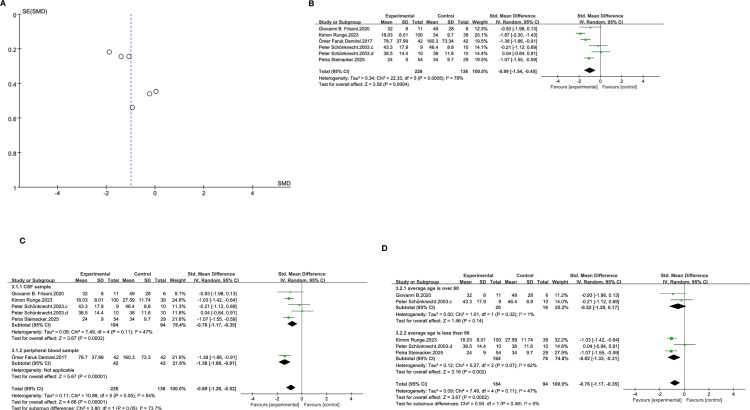
**(A)** Funnel plot for main analysis for p-tau and schizophrenia. **(B)** Forest plot of SMD and 95%CIs for p-tau and schizophrenia. **(C)** Forest plot of Std Mean Difference and 95% CIs for p-tau in cerebrospinal fluid of patients with schizophrenia. **(D)** Forest plot of Std Mean Difference and 95% CIs for p-tau in patients with average age over 60 and average less than 60.

The SMD between schizophrenia and phosphorylated tau protein is -1.07 (95%CI: [-1.55, -0.59]) (**Figure 3B**), it is statistically significant (p=0.0005). Peter’s experimental group was divided into those over 50 years old (c) and those under 50 years old (d). The funnel plot has poor symmetry (**Figure 3A**), indicating high heterogeneity. Considering that the differences in sample types are not significant (only Demirel’s study used peripheral blood samples, while the rest were cerebrospinal fluid samples), it might be attributed to differences in patients age or sample processing procedures. Based on this, a subgroup analysis of sample types and average age was conducted. It was not subjected to a separate subgroup analysis due to insufficient number of studies.

The SMD in the cerebrospinal fluid group is -0.76 (95% CI: [-1.17, -0.35], p=0.0002), and the peripheral blood subgroup is -1.38 (95%CI: [-1.86,-0.91]), both have statistical significance (**Figure 3C**), The SMD of the two samples was close, and there was less data on the peripheral blood subgroup, making it impossible to determine that sample factors were the source of heterogeneity. The results of the age subgroup analysis were similar to those of t-tau. The p-tau level in elderly patients was relatively normal (SMD=-0.52, 95%CI: [-1.20, 0.17], p=0.14). However, the proportion of younger patients was relatively lower than that of normal people (SMD=-0.82, 95%CI: [-1.33, -0.31], p=0.002), which might explain the source of heterogeneity (**Figure 3D**).

**Figure 4:**
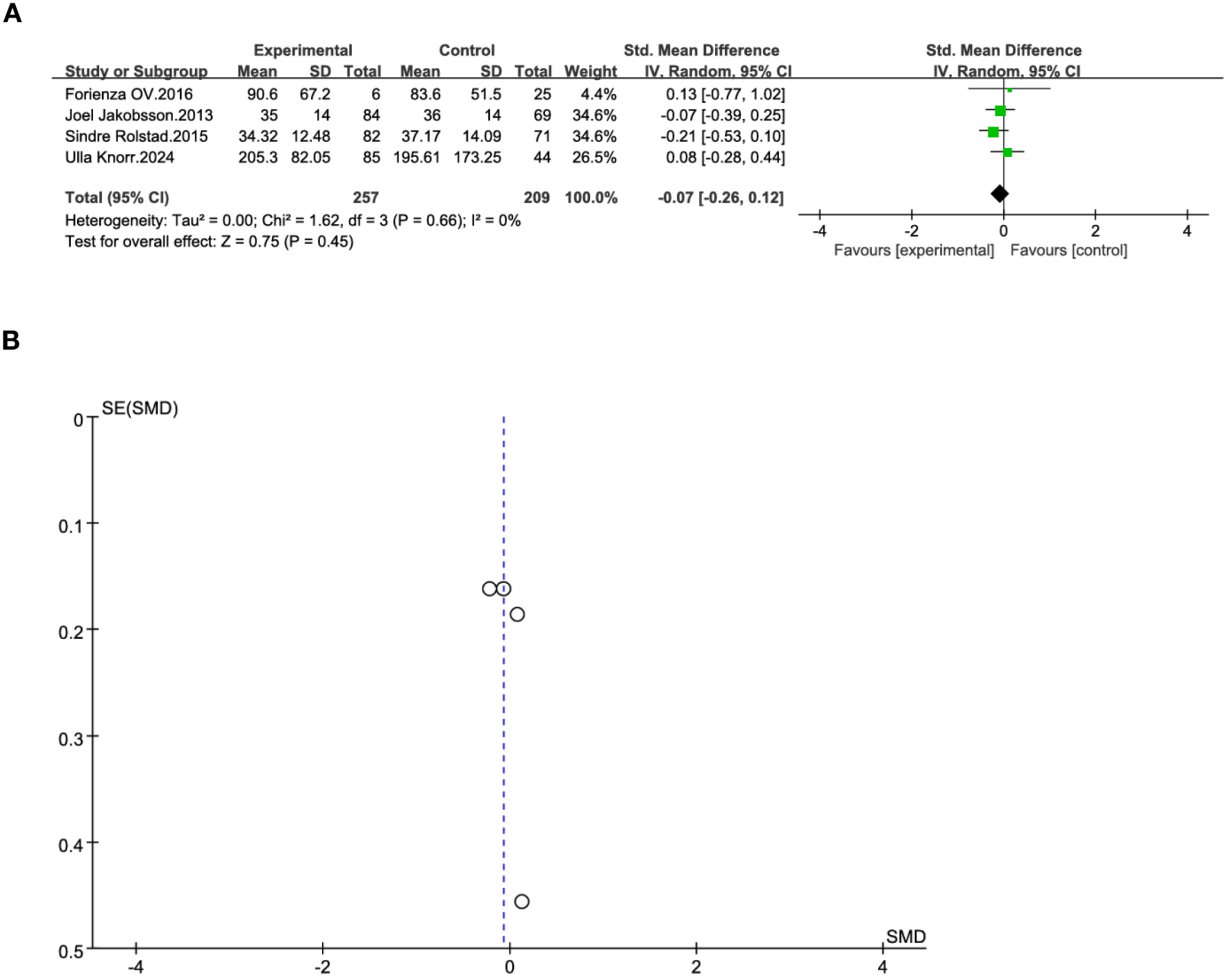
**(A)** Forest plot of SMD and 95%CIs for t-tau and bipolar disorder. **(B)** Funnel plot for main analysis for t-tau and bipolar disorder.

In the bipolar disorder term, we conducted a meta-analysis of four studies to explore the association between total tau protein concentration and BD. The diagnostic criteria for BD in the study were DSM-V and HAMD. Our meta-analysis found that the standardized mean difference between BD and tau protein was -0.07 (95% CI: [-0.26, 0.12], p=0.66) (**Figure 4A**), and the confidence interval included 0, indicating that the difference was not statistically significant. The I² value of 0 suggests low statistical heterogeneity, although this should be interpreted with caution given the small number of studies. In the funnel plot (**Figure 4B**), the asymmetrical distribution of the study points indicates the existence of heterogeneity.

## 4 Discussion

Our analysis indicates a consistent reduction in both tau protein and phosphorylated tau protein levels in schizophrenia, while tau levels do not appear to be significantly altered in BD. These findings suggest that the decreased total tau levels may compromise the stability of microtubules, thereby damaging the axons of neurons, causing abnormal synaptic connections and a decline in neuronal activities, thus increasing the risk of schizophrenia. Some studies have also pointed out that during the early stage of neural development, microtubules determine the migration of neurons, and low tau levels can inhibit neuronal migration, causing early migration disorders (10). The decreased level of phosphorylated tau in patients with schizophrenia may be due to the down-regulation of kinase activities such as GSK-3β (18). Insufficient phosphorylation of tau protein leads to the rigidity of the microtubule network, thereby disrupting the connections between neurons and resulting in the same outcome as a decrease in the total number of tau proteins. However, this specific process still requires more experiments to be confirmed.

Regarding the subgroup analysis of schizophrenia sample types, the changes in total tau protein in both cerebrospinal fluid and peripheral blood samples were statistically significant. However, CSF samples exhibited lower heterogeneity, a larger sample size, and more stable results, likely reflecting more suitable correspondence as clinical testing samples compared to peripheral blood. Some studies have pointed out that cerebrospinal fluid and peripheral blood have the same efficacy in diagnosing Alzheimer’s disease. Exosomes extracted from peripheral blood can cross the blood-brain barrier and spread in the brain (19). This conclusion may be used to guide the diagnosis of schizophrenia. Subgroup analysis of the age of schizophrenia patients revealed that both total tau protein and phosphorylated tau protein were statistically significant only in the younger patient subgroup. Considering that tau protein serves as a biomarker for Alzheimer’s disease, age may become an important factor influencing the changes in tau protein in schizophrenia patients. The abnormality of tau protein is related to neuronal microtubules, which corresponds to the significant changes in tau levels in the cerebrospinal fluid of patients with schizophrenia. However, both total tau level and phosphorylation level of schizophrenia patients show different changing trends from those of Alzheimer’s patients. Some studies have suggested that schizophrenia may have different pathogenic mechanisms from Alzheimer’s disease, and the neurofibrillary tangles in patients with Alzheimer’s disease are distinct (8).

Regarding the potential of tau as a biomarker for schizophrenia, although the observed changes are statistically significant, the high heterogeneity (partly attributable to different sample types) demonstrates that clinical application would require further validation through standardized and large-scale studies. This study still has certain limitations. For instance, the sample size is relatively small, and research in this field is currently scarce.

For patients with BD, tau protein cannot be used as a biomarker. Although studies have shown that both BD and Alzheimer’s disease are neurodegenerative disorders, their pathogenic mechanisms are different (20).

The MAPT gene in humans is located on chromosome 17, 17q21.1, and consists of 16 exons. It is responsible for expressing tau protein. tau proteinopathies are mostly caused by mutations in the MAPT gene, such as AI52T in Alzheimers disease (21), Schizophrenia may have a different tau pathogenic mechanism from Alzheimers disease. Some other studies have shown that changes in the 3R/4R-tau ratio and neurodegeneration caused by splicing dysregulation of tau exon 10 (22) have been observed in several tau protein diseases.

## 5 Conclusion

In summary, this meta-analysis provides evidence for reduced t-tau levels in patients with schizophrenia and offers guidance for future clinical trials. Given the current scarcity of biomarker research in schizophrenia, this work may serve as a foundation for significant future advancements. Given that the sample size was relatively small due to the inability to include more literature, we revised the conclusion by deleting and modifying the absolute terms.

## Data Availability

The original contributions presented in the study are included in the article/supplementary material. Further inquiries can be directed to the corresponding author.
